# Functional Properties of Kenaf Bast Fibre Anhydride Modification Enhancement with Bionanocarbon in Polymer Nanobiocomposites

**DOI:** 10.3390/polym13234211

**Published:** 2021-12-01

**Authors:** Samsul Rizal, Abdul Khalil H.P.S., E. M. Mistar, Niyi Gideon Olaiya, Umar Muksin, Marwan Marwan, Ikramullah, A. B. Suriani, C. K. Abdullah, Tata Alfatah

**Affiliations:** 1 Department of Mechanical Engineering, Universitas Syiah Kuala, Banda Aceh 23111, Indonesia; ikramullah@mhs.unsyiah.ac.id; 2 School of Industrial Technology, Universiti Sains Malaysia, Gelugor 11800, Penang, Malaysia; ck_abdullah@usm.my (C.K.A.); tataalfatah83@gmail.com (T.A.); 3 Nanotechnology Research Centre, Faculty of Science and Mathematics, Universiti Pendidikan Sultan Idris, Tanjung Malim 35900, Perak, Malaysia; absuriani@yahoo.com; 4 Department of Industrial and Production Engineering, Federal University of Technology, Akure PMB 704, Nigeria; ngolaiya@futa.edu.ng; 5 Department of Physics, Universitas Syiah Kuala, Banda Aceh 23111, Indonesia; muksin.umar@unsyiah.ac.id; 6 Department of Chemical Engineering, Universitas Syiah Kuala, Banda Aceh 23111, Indonesia; marwan@unsyiah.ac.id

**Keywords:** anhydride modification, Kenaf fibre, vinyl ester, bionanocomposites, bionanocarbon, reinforcing material

## Abstract

The miscibility between hydrophilic biofibre and hydrophobic matrix has been a challenge in developing polymer biocomposite. This study investigated the anhydride modification effect of propionic and succinic anhydrides on Kenaf fibre’s functional properties in vinyl ester bionanocomposites. Bionanocarbon from oil palm shell agricultural wastes enhanced nanofiller properties in the fibre-matrix interface via the resin transfer moulding technique. The succinylated fibre with the addition of the nanofiller in vinyl ester provided great improvement of the tensile, flexural, and impact strengths of 92.47 ± 1.19 MPa, 108.34 ± 1.40 MPa, and 8.94 ± 0.12 kJ m^−2^, respectively than the propionylated fibre. The physical, morphological, chemical structural, and thermal properties of bionanocomposites containing 3% bionanocarbon loading showed better enhancement properties. This enhancement was associated with the effect of the anhydride modification and the nanofiller’s homogeneity in bionanocarbon-Kenaf fibre-vinyl ester bonding. It appears that Kenaf fibre modified with propionic and succinic anhydrides incorporated with bionanocarbon can be successfully utilised as reinforcing materials in vinyl ester matrix.

## 1. Introduction

Composites with the incorporation of renewable biofibres dominate modern society since the products offer sustainable and eco-friendly characteristics [1]. Fibres are widely used in many fields [2,3], promoting the natural fibres to have a promising prospect as sustainable reinforcing material in composites [4]. Kenaf fibre, a biofibre, has been widely used for non-woven fibre mats. Its inherent beneficial properties, such as low-density with relatively high strength and thermal properties, corresponding to the promising bio-based material for reinforcing fibre in composites as an alternative to non-renewable fibre [5]. The utilisation of Kenaf fibres has increased the attention in composite application due to the biodegradability, non-toxicity and renewability features. As reported in the literature, approaches have been extensively devoted to developing Kenaf fibre as reinforcing fibre in the composite product [6]. Prakash and Viswanthan [7] used Kenaf fibre as a reinforcing material in fabricating composite and reported enhanced mechanical properties and thermal stability relative to unreinforced composite. Chee et al. [8] investigated the physical properties of nanocomposites reinforced with Kenaf fibre and nanofiller. They reported that combining the fibre and nanofiller could improve the water barrier and dimensional stability of composites at 56% and 37%, respectively. Interfacial interaction of fibre-matrix in the composite structure effects in functional properties of the bionanocomposites.

The intrinsic moisture absorption characteristic of biofibre is the main disadvantage in the application as a reinforcement material [9]. Poor biocomposite functional properties triggered by low hydrophilic biofibre with hydrophobic matrix remains a challenge. Improving the structure and chemistry of cell walls can be achieved through surface modification to improve biocomposites’ functional properties [10]. Cervantes et al. [11] chemically modified agave biofibre using propionic anhydride could improve the biocomposites’ compatibility. The flexural strength and water barrier of biocomposites with propionylated fibre improved up to 170% and 35% compared to uncompatibilised biocomposites. In another study, Ahmed et al. [12] modified jute fibre using succinic anhydride as reinforcing fibre in polypropylene biocomposites. The strength, hardness and water barrier properties were enhanced due to the enhanced interface adhesion by the anhydride modification on the biofibre. Hence, using propionic and succinic anhydrides modification of Kenaf fibre is essential to improve the hydrophobicity and compatibility in vinyl ester matrix interaction that can enhance the biocomposites’ functional properties.

Nanofiller can enhance the mechanical strength and thermal stability of nanocomposite [13]. Bionanocarbon presents ideal properties in the composite application as it has low density, great thermal and strength properties [14]. Recently, Nikolaeva et al. [15] reported using nanocarbon filler in the preparation of nanocomposites where the functional performance of the obtained composite can be substantially improved. Nano-scale size and negative surface charge of biocarbon make the reinforcing filler more dispersive in a cationic matrix-like vinyl ester [16]. Researchers have investigated agricultural wastes as a precursor in biocarbon production to reduce the overdependence on wood-based plants [17,18].

On the other hand, vinyl ester has been widely utilised for liquid-moulding formulations due to its low weight, good chemical resistance, low cost, high toughness, low volume shrinkage, and low exothermal heat [19]. Consequently, it has been employed in many industrial-scale applications such as coatings, adhesives, structural laminates, military/aerospace, surface vehicles, moulding compounds, and automobile parts [20]. However, a neat composite of the vinyl ester is brittle. The incorporation of Kenaf fibre and bionanocarbon in vinyl ester can be ideal for strengthening and cost-reducing the matrix [21]. Nevertheless, the hydrophilicity and impurities on Kenaf fibre decrease the interfacial bonding in the matrix. A good approach to solve the challenge is using anhydride modification of the biofibre that can improve the compatibility of composite.

In light of the issues mentioned above, the present work was focused on fabrication and characterisation of vinyl ester bionanocomposites reinforced with anhydrides modified Kenaf fibre and the enhancement characteristics by incorporating bionanocarbon as nanofiller. The obtained bionanocomposites were characterised to study the physical, chemical resistance, thermal, structural, mechanical, and morphological characteristics as functional properties to determine the effect of the anhydrides modified Kenaf fibre and bionanocarbon in bionanocomposites structure. For the first time, bionanocomposites reinforced with propionylated and succinylated Kenaf fibres with the incorporation of the novel bionanocarbon is studied. The present work comprehensively investigates functional properties, including the anhydride modified fibres, bionanocarbon, and bionanocomposites. Hence, the interest of material scientists and researchers in all related fields will increase, and the field of nanobiocomposite will advance.

## 2. Materials and Method

### 2.1. Materials

Nonwoven Kenaf fibre mat was supplied from Lembaga Kenaf dan Tembakau Negara, Kota Bharu, Kelantan, Malaysia. The thickness of Kenaf fibre used was 10 mm. OPS chips from Ulu Keratong palm oil mill, Segamat, Johor, Malaysia, were employed as raw material for bionanocarbon isolation. Vinyl ester with 42% styrene monomer content and methyl ethyl ketone peroxide or MEKP (C_8_H_18_O_6_) were purchased from Zarm Scientific & Supplies Sdn. Bhd., Malaysia. Succinic anhydride (C_4_H_4_O_3_ ≥ 99.0%), propionic anhydride (C_6_H_10_O_3_ ≥ 99.0%), N,N-dimethylformamide (C_3_H_7_NO ≥ 99.8%), sodium formate (HCOONa ≥ 99.0%), toluene (C_7_H_8_ ≥ 99.5%), ethanol (C_2_H_5_OH 99.8%), acetone (C_3_H_6_O ≥ 99.5%), sodium hydroxide (NaOH ≥ 97.0%), hydrochloric acid (HCl 37.0%), benzene (C_6_H_6_ ≥ 99.8%), carbon tetrachloride (CCL_4_ ≥ 99.9%), nitric acid (HNO_3_ 70.0%), acetic acid (CH_3_COOH ≥ 99.0%), sodium carbonate (Na_2_CO_3_ ≥ 99.9%), ammonium hydroxide (NH_4_OH 28.0%), and cobalt naphthenate (CoC_22_H_14_O_4_ 6.0%) were obtained from Sigma Aldrich, St. Louis, MO, USA. All chemicals are analytical grade.

### 2.2. Anhydride Modification of Nonwoven Kenaf Fibre

**Figure 1:**
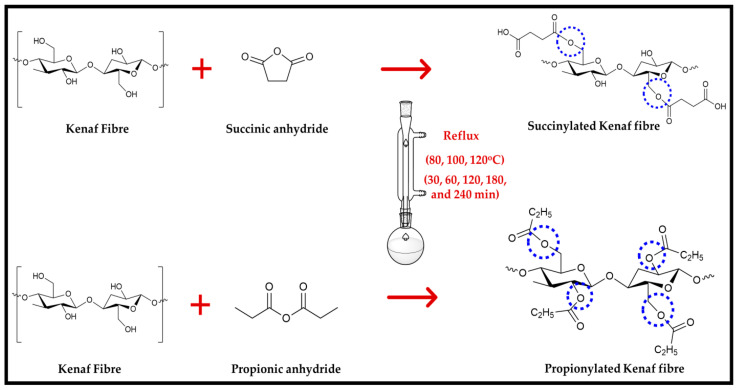
Schematic reaction of anhydride modification on Kenaf fibre.

The Kenaf fibre mats with a dimension of 200 mm × 200 mm × 5.5 mm were put in a soxhlet apparatus. Toluene: acetone: ethanol: with ratio (*v*/*v*) of 4:1:1 for 3 h was applied to prepare the extractive-free fibres. The specimens were then put in an oven overnight at 110 °C for the drying process. The biofibres were placed in a vacuum desiccator for 2 h over silica gel before weighing. The biofibres (5 sets of each experiment) were transferred to a flask containing a solution of the propionic anhydride or succinic anhydride in N-dimethylformamide in the presence of the catalyst sodium formate (10:1). The anhydrides modification process adopted three temperature reaction settings of 80, 100, and 120 °C with five residence time periods of 30, 60, 120, 180, and 240 min [22,23]. At the end of the modification process, the flasks were lifted out from the heater, and the hot reagent was poured. The dry ethanol was used to rinse the obtained fibres surface to expunge the remaining acid. Before oven drying at 80 °C for overnight, the specimens were open air-dried for another 2 h and weighed. The schematic reaction of anhydrides modified Kenaf fibre is presented in Figure 1.

### 2.3. Preparation of Bionanocarbon

Bionanocarbon was produced from oil palm agricultural waste from the oil palm mill industry via one-step NaOH activation. The raw material was first cleaned with distilled water and then opened air-dried for 24 h. The dried OPS chips were crushed using a grinder with a 1.0 mm filter hole. A Retsch mill with a filter of 0.25 mm was then used to grind the OPS particles further. The obtained particles were sieved using 25 µm sieve size and were oven-dried at 110 °C for 24 h. The precursor was then mixed in NaOH solution using an impregnation ratio of 1:0.5 (mass of OPS to NaOH. The mixture was oven-dried at 120 °C for 4 h followed by activation at 700 °C for 30 min in a muffle furnace [24,25]. The obtained biocarbon was transferred to a desiccator. After reaching room temperature, the carbon material was washed using HCl 0.1 M at 85 °C [26]. Afterwards, hot distilled water was used to rinse the biocarbon until pH ∼ 7 [27]. The biocarbon was oven-dried at 110 °C for overnight. Nanosize of biocarbon was fabricated through high-energy ball milling at a rotational speed of 170 rpm for 24 h. A ratio of ball to the biocarbon particle of 10:1 (*w*/*w*) was placed in the stainless-steel chamber. Finally, the bionanocarbon was oven-dried at 110 °C for 24 h and kept inside the desiccator in a glass vial.

### 2.4. Characterisation of Kenaf Fibre and Bionanocarbon

The functional properties of the unmodified, anhydrides modified fibre, bionanocarbon and nanobiocomposites were investigated for the characterisation studies. The fibre specimens’ weight percent gain (WPG) after the modification process was determined according to the consumption of propionic and succinic anhydrides onto non-woven kenaf fibre mats. As shown in Equation (1), weight gain was calculated from the percentage of the weight of the anhydrides modified fibres. Each experiment was performed five times, and the results were finalised according to the average value with the standard deviations. The sessile drop method on KSC CAM 101 (KSV Instruments Ltd., Espoo, Finland) was used to observe the contact angle of unmodified and anhydrides modified non-woven kenaf fibres. Meanwhile, a FESEM (FEI Quanta FEG 650, Thermo Fisher Scientific, Eindhoven, The Netherlands) investigated the fibre morphologies. The chemical structure functional group of fibres and bionanocarbon were observed by an FT-IR Prestige-21 spectrophotometer (Shimadzu, Chiyoda-ku, Tokyo, Japan). To determine the thermogravimetric analysis, a Mettler-Toledo thermogravimetric analyser model TGA/DSC 1 (Mettler Toledo, Schwarzenbach, Switzerland) was used to determine the thermogravimetric analysis (TGA) of the fibre and bionanocarbon samples. The morphological properties of bionanocarbon were analysed via transmission electron microscopy (TEM), energy-filtered EFTEM Libra 120-Carl Zeiss instrument (Oberkochen, Germany). Particle size and zeta potential measurements of bionanocarbon were measured using dynamic light scattering (DLS) on a Malvern Zetasizer Nano ZS Ver. 7.11 (Malvern Instruments, Malvern, UK).

(1) $$\text{Weight percent gain}\;(\%)=\frac{\text{Weight gain}}{\text{Original weight}}\;\times \;100$$

### 2.5. Preparation of Nanobiocomposites

The anhydrides modified Kenaf fibres at the optimum WPG obtained from 100 °C of temperature reaction and 180 min of retention time were utilised as reinforcing materials to prepare nanobiocomposites. The nanobiocomposites of vinyl ester reinforced by unmodified or anhydrides modified kenaf fibres (fixed at 40 wt% of fibres loading) were introduced with 0, 1, 3, 5 wt% of bionanocarbon (parts per hundred resin) [28]. As a catalyst and accelerator, approximately 1.5 wt% of MEKP and 0.2 wt% of cobalt naphthenate were utilised to fabricate nanobiocomposites [29]. The neat vinyl ester composite and nanobiocomposites reinforced by unmodified, propionylated and succinylated Kenaf fibres were loaded with 0, 1, 3, and 5 wt% of bionanocarbon and were tagged as VE, VE/UK, VE/PK/NC0, VE/PK/NC1, VE/PK/NC3, VE/PK/NC5, VE/SK/NC0, VE/SK/NC1, VE/SK/NC3, VE/SK/NC5, respectively. The vinyl ester-hardener mixture was compounded with the nanofiller and homogenised using a mechanical stirrer at 1000 rpm for 10 min.

The mixture was moulded with a Hypaject Mark II RTM injection system (Plastech, Thermoset Tectonics, Gunnislake, UK) equipped with a 200 mm × 200 mm × 5.5 mm mould containing the fibre. Prior to the curing process, the metal mould was coated with a thin layer of silicon oil solution as a releasing agent. The mixture was transferred into the mould at 200 kPa pressure and ambient temperature via vacuum assistance. A vacuum pump was connected in the vent hole of the mould to improve the air evacuation and to avoid bubble trapping in the mixture. The obtained composites were cured overnight at room temperature and then post-cured in an oven for another 4 h at 80 °C. The composites were then placed in a desiccator containing granulated silica gel until room temperature was achieved. Afterwards, they were cut to test samples and were put in a zip lock bag and stored in a desiccator for analysis.

### 2.6. Characterisation of Bionanocomposites

The characterisation analysis studied the functional properties of the prepared composites. The physical characteristics were determined by the density, void content, and chemical resistance analysis. The composite density was determined by measuring the mass (*m*) and thickness (*t*) of 2 cm × 2 cm cut samples according to ASTM D 1895. Each experiment was performed five times, and the results were finalised according to the average value with the standard deviations. It was estimated by Equation (2).

(2) $$\text{Density}\;(\rho )=\frac{m}{V}$$

where *V* = *l* × *b* × *t* is the volume of the cut samples.

The composites void content was evaluated, ASTM D 2734 procedure was applied. Each experiment was performed five times, and the results were finalised according to the average value with the standard deviations. It was defined from the experimental and theoretical densities of the composites by Equation (3) [30].

(3) $$\text{Void content}\;(\%)=\frac{\rho_{\mathrm{theoretical}}-\rho_{\mathrm{experimental}}}{\rho_{\mathrm{theoretical}}}\;\times \;100$$

where

$$\rho_{\text{theoretical}}=\frac{1}{\left[\frac{W_{f}}{\rho_{f}}+\frac{W_{fb}}{\rho_{fb}}+\frac{W_{m}}{\rho_{m}}\right]}$$

*W_f_* is the filler weight fraction, *W_fb_* is the fibre weight fraction, *W_m_* is the matrix weight fraction, *ρ_f_* is the filler density, *ρ_fb_* is the fibre density, and *ρ_m_* is the matrix density.

The chemical resistance characteristic of the prepared bionanocomposites was studied using the ASTM D543-87 standard. Studies were conducted to investigate the effect of solvents, acids, and alkalis on the bio nanocomposite. The investigated solvents, acids, and alkalis were (C_6_H_6_, C_7_H_8_, and CCL_4_), (HCl, HNO_3_, and CH_3_COOH), and (NaOH, Na_2_CO_3_, NH_4_OH), respectively. Five pre-weighed samples in each case were immersed in the respective chemical solutions for 24 h. The samples were removed and washed using distilled water and then dried by pressing the specimens on all sides with a filter paper (accuracy in ± 0.01 mg). Each experiment was performed five times, and the results were finalised according to the average value with the standard deviations. The percentage weight gain/loss was determined using the following Equation (4):

(4) $$\text{Weight gain/loss}\;(\%)=\frac{\mathrm{Final}\;\mathrm{weight}\;-\;\mathrm{Original}\;\mathrm{weight}}{\mathrm{Original}\;\mathrm{weight}}\;\times \;100$$

The mechanical characteristics of the nanobiocomposites were evaluated in terms of tensile, flexural, and impact properties. ASTM D638 standard was implemented to determine the tensile strength, tensile modulus, and elongation at the break of the obtained composites. Meanwhile, the ASTM D790 standard was applied to investigate the flexural strength and modulus characteristics. The flexural and tensile tests were performed through an INSTRON 5582 universal testing machine (Norwood, MA, USA). ASTM D256 procedure for Izod analysis was carried out to measure the impact strength. The composite specimens were analysed using a Gotech testing instrument, Model GT-7045 MD (Taichung City, Taiwan). Five test samples were evaluated for each specimen composition, and the average values were recorded with standard deviations.

Morphological analysis after the tensile test was evaluated by FESEM (FEI Quanta FEG 650, Thermo Fisher Scientific, Eindhoven, The Netherlands). Thin specimens were assembled on an aluminium (Al) stub holder with double-sided copper (Cu) tape holder. The tensile fracture samples’ electrical conductivity was enhanced by coating with a platinum (Pt) layer through sputter and coater Quorum Technologies Q150T. Under conventional secondary electron imaging stipulations, the FESEM images were observed at an accelerating voltage of 10 kV.

Structural analysis of the nanobiocomposites was evaluated using the Fourier transformation infrared (FT-IR). The specimen analysis was determined using an FT-IR Prestige-21 spectrophotometer (Shimadzu, Chiyoda-ku, Tokyo, Japan). Before analysis, the specimens were prepared and dried at 60 °C in an oven for 24 h. Before it was placed in the FT-IR instrument and the transmittance was achieved, the powder sample was mixed with KBR and was pressed into the circular film. The FT-IR analysis was performed at 4 cm^−1,^ and the wavenumber ranged from 4000–400 cm^−1^.

Thermal characteristics of the nanobiocomposites were evaluated by Mettler-Toledo thermogravimetric analyser model TGA (Mettler Toledo, Schwarzenbach, Switzerland). With a reference (a pre-weighed empty alumina crucible), approximately 10 mg of nanobiocomposite sample was weighed in an alumina crucible and was placed in a thermogravimetric analyser. The thermal test under the flowing nitrogen (N_2_) at a 50 mL/min flow rate was carried out within furnace temperature range from 30 °C to 800 °C with a heating rate of 10 °C/min. Finally, the obtained results were derived using the STAR^e^ SW 10.00 software program for the investigation of the mass loss (%), initial degradation temperature (T_on_), and maximum degradation temperature (T_max_).

## 3. Results and Discussion

### 3.1. Characterisation of Nonwoven Kenaf Fibre

#### 3.1.1. Weight Percent Gain Properties

**Figure 2:**
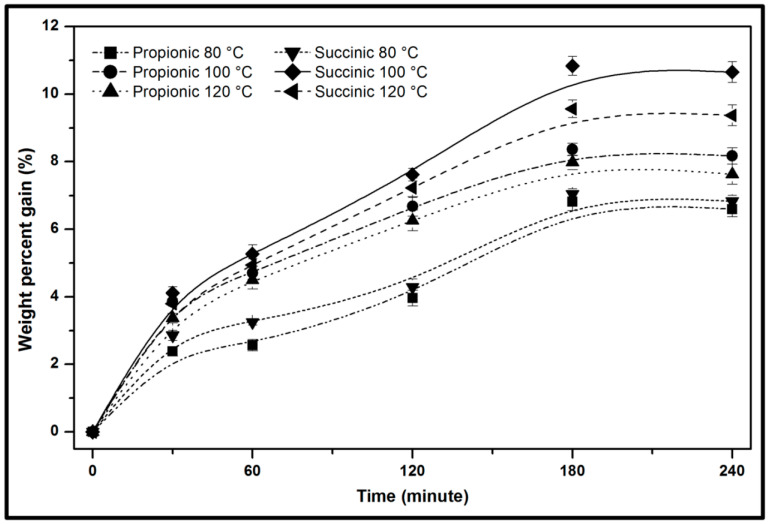
Weight percent gain of anhydride modification on Kenaf fibre with different reaction temperatures and different retention times.

The weight percent gain of Kenaf fibre was influenced by the reaction temperature, retention time, and anhydride modification. The Kenaf fibre showed substantial weight gain as time rose at the initial step until saturation was obtained regardless of the divergence in temperature, as presented in Figure 2. This showed that the fibre swelling may impede interfacial bonding beyond 180 min and may probably result in cracking at the adjoining matrix [31]. Increasing the WPG was obtained at the initial step owing to the relative reaction of functional hydroxyl groups and the diffusion rate of reagents in the interface of fibre-matrix as shown in the reaction profile [32]. The WPG increased as the reaction temperature and reaction time increased.

Nevertheless, a further increase in the reaction temperature at 120 °C showed a decrease in the WPG [33]. As can be seen, spontaneous weight gain was obtained beyond which the Kenaf fibre depicted a steep decrease in WPG as the reaction time was increased from 60 min to 180 min. The decrease in WPG due to increased reaction temperature could be affected by the decomposition of the fibre’s cellulose, hemicellulose, and lignin [34]. Hence, the optimum reaction temperature of Kenaf fibres should not be exceedingly high and should appropriately permit rapid reaction to minimise the possibility of fibre decomposition. A reaction temperature at 100 °C provided an optimum weight gain as exhibited by the kenaf fibre. According to the WPG results, Kenaf fibre modified using succinic anhydride showed higher WPG (10.84%) compared to fibre modified with propionic anhydride (8.36%). The reason was probably owing to the adduction of a 4-carbon chain with the carboxyl group to the succinylated fibres, whereas propionylation adducted only a 3-carbon hydrophobic group during modification. Succinylation can swell the fibre structure more effectively, making reactive chemicals sites more accessible and enhancing the reaction rate of modification, which results in higher WPG.

#### 3.1.2. Morphological Analysis

**Figure 3:**
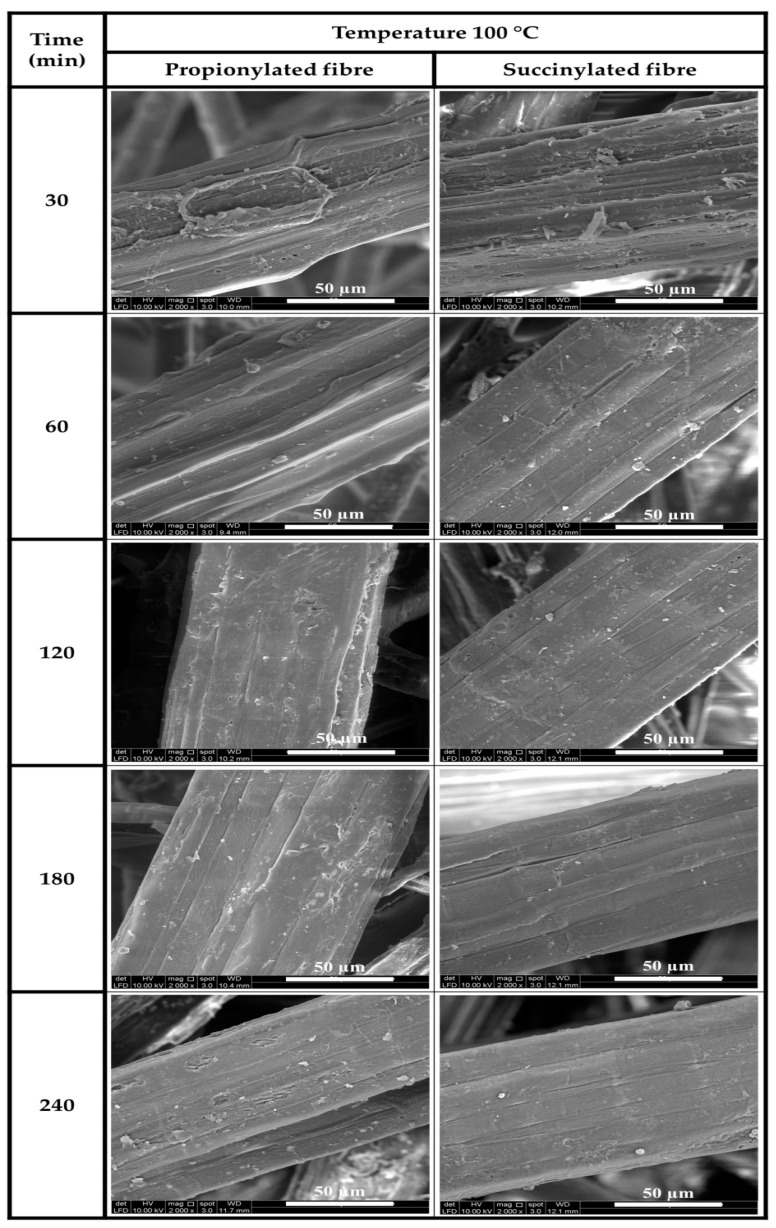
SEM micrograph of anhydride modification on Kenaf fibre at 100 °C with different retention times.

The fibre surface morphology of propionic anhydride and succinic anhydride modifications onto Kenaf fibre at 100 °C with different retention times is displayed in Figure 3. From the SEM images, succinylated fibres provided a smoother surface than propionylated fibres owing to the existence of carboxylic groups resulting from the succinic anhydride modification, which can react with waxy substances from fibres without producing by-products [31]. This indicated that the modification results in a much smoother surface due to removing surface impurities that block reactive chemical groups [35]. The Kenaf fibre improved the textural surface properties of the fibre as retention time increased. This indicated that the presence of wax, oil, and other impurities decreased as the temperature increased as the reaction period increased [36]. Such impurities impeded the interaction of fibre-matrix bonding. An enhanced surface as the retention time increased to 180 min showed greater interaction at the interfacial boundary of the reinforcing materials [23]. This was probably due to the decrease of non-cellulosic materials, silica, inorganic compounds and enhanced compatibility within the fibre-matrix contexture. As a result, improved void spaces were obtained to support the functional properties as reinforcing fibre [37]. The effect of succinic anhydride modification of Kenaf fibre and the impurities removal rendered better enhancement in surface morphology of fibre and improved the adhesion of fibre-matrix interface.

#### 3.1.3. Structural, Wettability, and Thermal Properties

**Figure 4:**
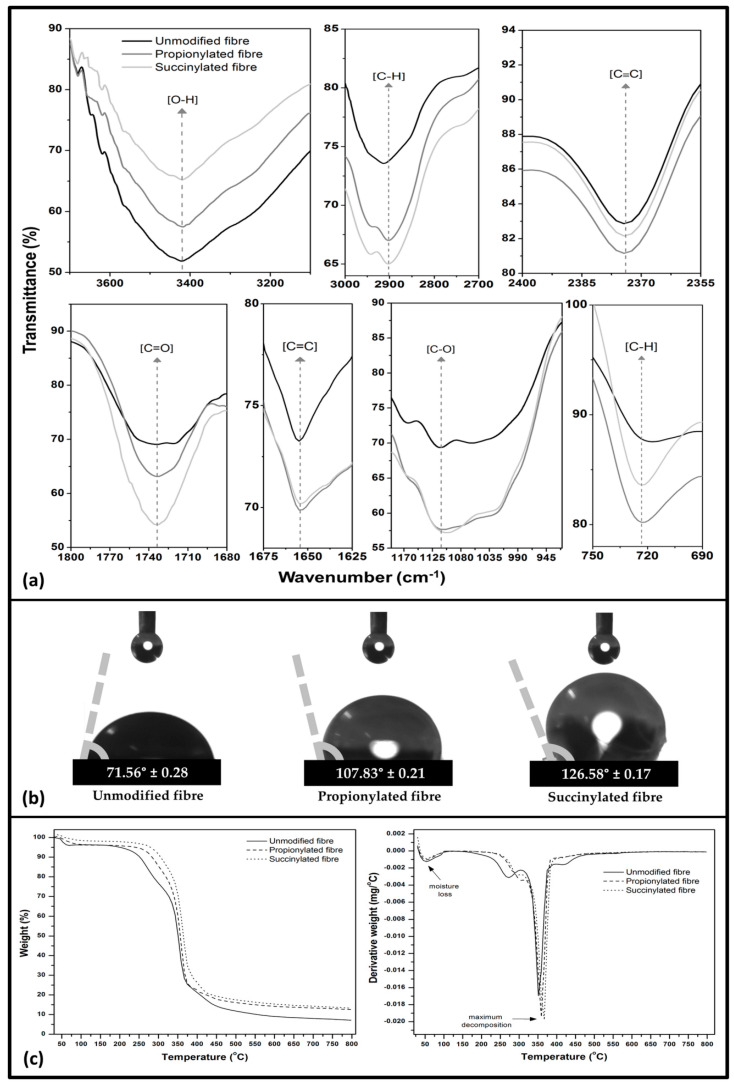
Characterisation of unmodified and anhydrides modified Kenaf fibre (**a**) FT-IR, (**b**) contact angle, and (**c**) thermal analysis.

Figure 4 depicts chemical structural, contact angle, and thermal analysis of the unmodified and anhydrides modified Kenaf fibre. As seen in Figure 4a, a high intensity was identified about 3200–3600 cm^−1^ for all samples indicating the O-H (hydroxyl) groups stretching vibration. The effect of anhydride modification was identified as the fibre showed a reduction of hydroxyl groups compared to unmodified fibre, indicating an increase in the hydrophobicity properties. However, succinylated fibre provided the lowest transmittance spectra of the hydroxyl groups. A band region for all fibre samples signified the presence of C–H (methyl) groups at around 2700–3000 cm^−1^ and 700–750 cm^−1^, while the presence of C≡C stretching vibration in alkynes groups appeared in the signal region of 2350–2400 cm^−1^. The intensities of 1650–1800 cm^−1^ and 1625–1675 cm^−1^ were attributed to C=O (carbonyl) groups and C=C (carboxyl) groups, respectively. A peak region of 950–1150 cm^−1^ was associated with C–O (alkoxy) groups. According to the FT-IR result, the anhydrides were successfully modified the Kenaf fibre’s chemical structure, particularly as indicated a decrease of cellulosic hydroxyl groups to increase the carbonyl and methyl groups [38].

The effect of anhydrides modification in the wettability properties based on the contact angle test was exhibited in Figure 4b. Both anhydrides modified fibres showed an enhancement in contact angle than fibre without modification. Succinylated fibre had the highest contact angle of 126.58° ± 0.17, while propionylated showed 107.83° ± 0.21. Mohammed et al. [33] reported that anhydride-modified fibres become hydrophobic (contact angle > 90°) due to the introduction of carboxyl and dispersive aliphatic chain (–CH2–CH3) groups on the modified fibre surface, resulting in superior compatibility between the fibre and matrix with fewer voids. These functional groups had the probability of indirectly affecting the wetting properties on the fibre surface. Such improvement in hydrophobicity of fibre could improve the interfacial bonding interaction with the vinyl ester as a hydrophobic polymer matrix.

Thermal analysis of unmodified and anhydrides modified fibres are shown in Figure 4c. Evaporation of absorbed moisture was observed at initial temperature to 100 °C for all specimens. This was attributed to the fibre humidity even though the hydrophilic nature of the fibre hindered total moisture loss because of structurally linked molecules in the fibre structure [39]. The first stage of degradation occurred around endothermic 250 °C–300 °C. Anhydride modified fibres presented a lesser weight loss relative to unmodified fibre. This was probably due to the decreased hydrophilicity of the fibre by the anhydrides modification. The maximum weight loss was observed at the second stage of degradation at a temperature between 350 °C and 400 °C. At this stage, volatilisation of all fibre specimens was observed in the DTG curve. The T_on_ shifted from 328.94 °C (unmodified) to 343.17 °C (propionylated), and 358.39 °C (succinylated) and T_max_ shifted from 352.05 °C to 361.38 °C and 374.51 °C, respectively. In conclusion, the anhydrides modification could enhance the thermal stability of the fibre structure, which is a beneficial factor as a composite component. Interfacial interaction of anhydride groups with hydrophilic substances on the fibre was responsible for the higher initial and maximum degradation temperatures [40].

### 3.2. Characterisation of Bionanocarbon and Bionanocomposites

#### 3.2.1. Characterisation of Bionanocarbon

**Figure 5:**
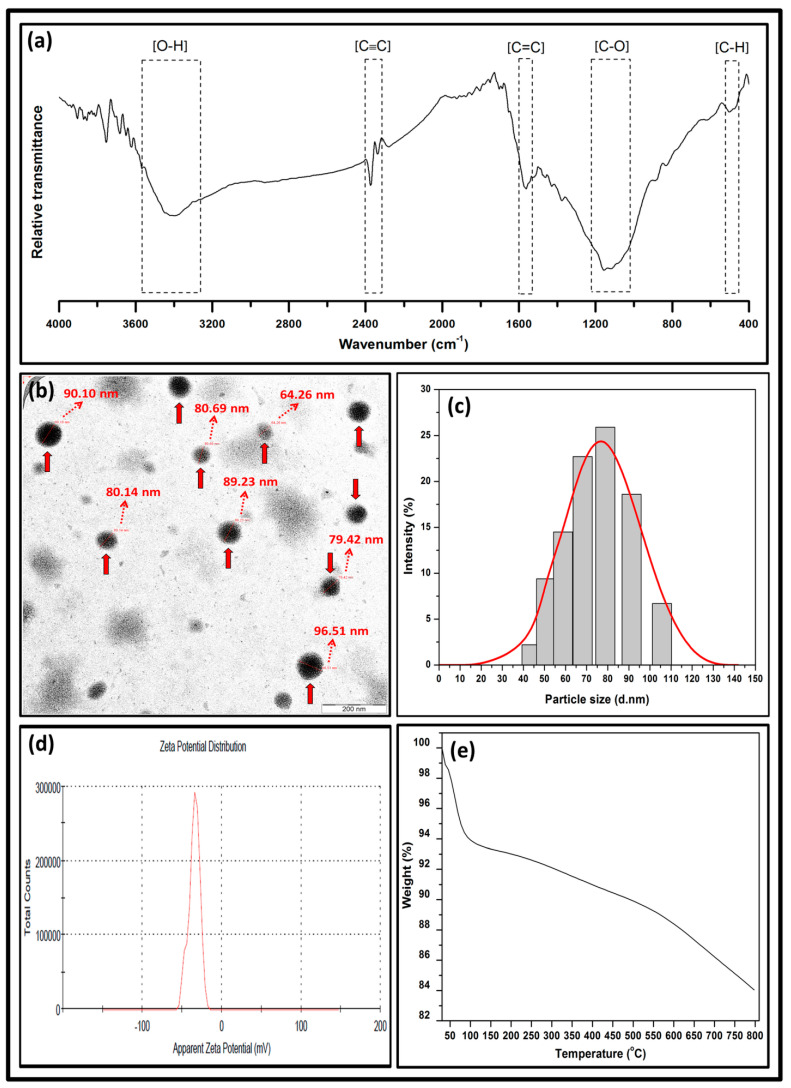
Characterisation of bionanocarbon (**a**) FT-IR analysis, (**b**) TEM, (**c**) particle size distribution, (**d**) zeta potential distribution, and (**e**) TGA.

Figure 5 exhibits the chemical structural, morphological, particle size distribution, zeta potential, and thermal properties of the produced bionanocarbon. Figure 5a illustrates the FT-IR transmittance spectra of the bionanocarbon. A broad region band around 3300–3600 cm^−1^ was associated with O–H (hydroxyl) groups. The transmittance region of 2325–2400 cm^−1^ revealed the presence of C≡C (alkynes). A region peak at 1550–1600 cm^−1^ indicated C=C (carboxyl). An intensity region of 1000–1200 cm^−1^ indicated the presence of C=C (carboxyl). A signal region of 1050–1150 cm^−1^ depicts the presence of C–O (alkoxy), while the presence of asymmetric C–H (methyl) stretching vibration in methyl groups emerged in the transmittance region of 450–500 cm^−1^. A similar finding on bionanocarbon functional group properties was also reported by Fan et al. [41].

The bionanocarbon’s morphological properties showed uniformly dispersed particle sizes ranging from 64.26 nm–96.51 nm in Figure 5b. Black colouration patches across the boundary were obtained. The micrograph of the bionanocarbon presented a great level of distribution. This uniformly dispersed particle size of the nanofiller could improve the compatibility between the fibre-matrix network. It could potentially charge the voids and gaps within the interfacial interaction, which increased the functional properties of the nanobiocomposite [42]. The particle size distribution and zeta potential were illustrated in Figure 5c,d, respectively. It was denoted in Figure 5c that the particle diameter of the bionanocarbon was in the range of 44 nm to 106 nm, with an average particle size of 74.18 nm. A trend of increase was indicated in particle diameter as the percentage intensity increases, although there was a steep decrease in particle size diameter within 100 nm to 110 nm. As shown in Figure 5d, the zeta potential value of bionanocarbon was −34.7 ± 3.95 mV. The high estimated negative value could be attributed to the significance of the carboxyl functional group on the surface of the bionanocarbon [43]. The increase in surface densities of the functional groups such as hydroxyl and carboxyl is significant for the filler’s miscibility, compatibility, and dispersion stability in the matrix [44,45]. The zeta potential below −30 mV indicated the stability of the surface charge of the bionanocarbon particles, which implies that sufficient mutual repulsion resulted in colloidal or emulsion stability [46].

Figure 5e depicts the percent weight loss of bionanocarbon as a function of temperature. The TGA analysis showed an immensely slight mass loss of bionanocarbon. The first weight loss from 100 to 150 °C was associated with removing free hydrogen-bonded water molecules [47,48]. The initial weight loss was followed by the main weight loss at an initial decomposition temperature of 579.58 °C. The oxygen functional groups on the bionanocarbon surface were converted to oxides of carbon and were relieved from the bionanocarbon in gaseous form at this stage upon degradation [49]. During the heating, the reduction of hydroxyl groups and secondary reaction with adjacent carboxyl groups occurred. A total yield of 83.99% was achieved at 800 °C, indicating a promising nanomaterial in thermal resistance.

#### 3.2.2. Characterisation of Bionanocomposites

##### Chemical Structural Analysis

**Figure 6:**
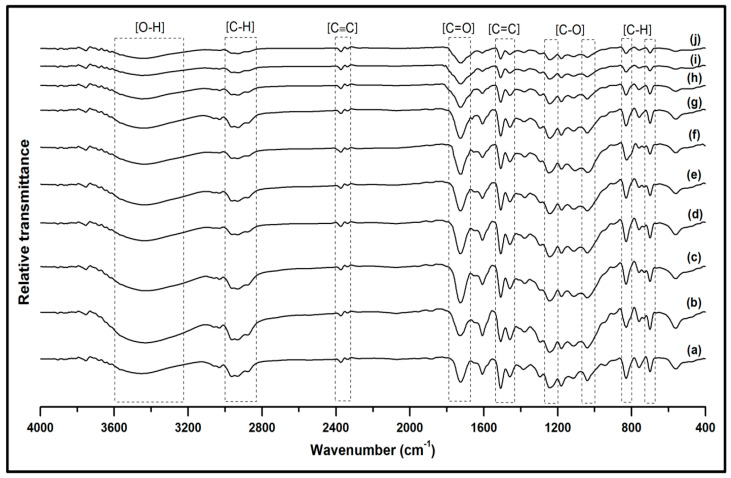
FT-IR analysis of (**a**) VE, (**b**) VE/UK, (**c**) VE/PK/NC0, (**d**) VE/PK/NC1, (**e**) VE/PK/NC3, (**f**), VE/PK/NC5, (**g**) VE/SK/NC0, (**h**)VE/SK/NC1, (**i**) VE/SK/NC3, and (**j**) VE/SK/NC5.

Functional groups from the FT-IR transmission spectra in Figure 6 were evaluated to determine the chemical structures of the neat, unmodified fibre, propionylated fibre, and succinylated fibre reinforced composites. The broad region at 3200–3600 cm^−1^ was associated with the stretching vibration of the O–H (hydroxyl) groups. A band region at 2850–3000 cm^−1^ was ascribed to the asymmetric C–H representing stretching vibration in methyl groups. A region peak of about 2325–2400 cm^−1^ showed the presence of C≡C (alkynes) groups. The increase in stretching bands around 1700–1800 cm^−1^ was ascribed to C=O (carbonyl) groups vibration that indicated increased interaction of the hydroxyl groups in the cellulosic Kenaf fibres with anhydrides modification process [38]. A region peak of about 1450–1550 cm^−1^ was the signal for C=C (carboxyl) groups. The region transmittance of 750 and 850 cm^−1^ indicated C–O (alkoxy) groups stretching vibration. According to the FT-IR analysis, the transmittance spectra of the bionanocarbon, unmodified, propionylated, succinylated Kenaf fibre and the obtained composites showed that the active functional groups influenced the compatibility of the bionanocarbon-fibre-matrix network. However, no different functional group appeared other than those present in the component material. It shows that a considerable increase in the properties because of the interfacial adhesion provided by the nanofiller and the binding properties of the reinforcing fibre and matrix. This defines the miscibility and interfacial interaction between the three constituents.

##### Physical Properties

**Figure 7:**
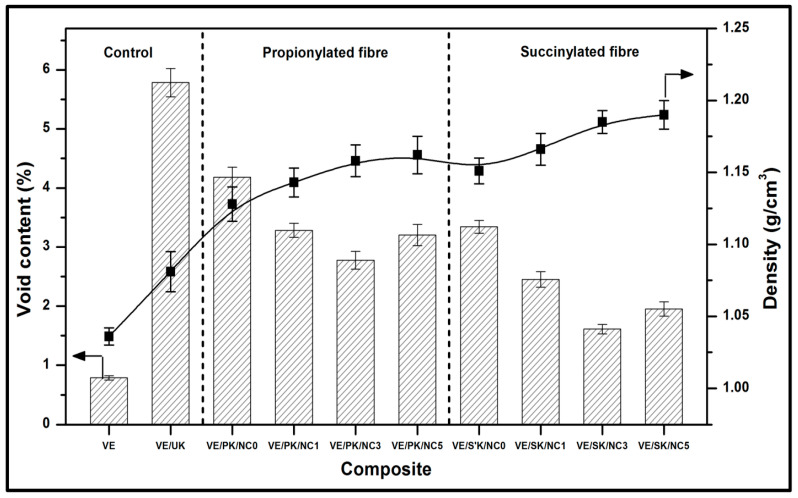
Void content and density of nanocomposite.

Void content and density of obtained composites are displayed in Figure 7. Void content plays a vital role in the physical properties of composites as low void content is beneficial to achieve optimised functional properties of the composites. The presence of void content in the composites considerably decreases the properties of the composites. The void content in the obtained composites were in the range of 0.788–5.784%. The significance of the succinic derived kenaf nanocomposite provided indicated better compatibility between fibre and matrix compared to the propionic kenaf-based nanocomposite. High cross-linking between succinylated Kenaf fibre and vinyl ester may be the greater aspect in reducing void content [50]. Also, the void content in the composites was reduced with the incorporation of bionanocarbon loading, which indicates that the bionanocarbon enhanced interfacial interaction of the bionanocomposite structure. Its homogeneous dispersion corresponds to enhancing the nanofiller-fibre-matrix interaction [51].

On the other hand, the density of obtained composites depends on the relative proportion of matrix, reinforcement material and nano-structures filler. The neat VE had the lowest density (1.036 g cm^−3^), while succinylated fibre bionanocomposite with 5% bionanocarbon showed the highest density (1.190 g cm^−3^). This was probably due to the reduction in void content after modification of Kenaf fibre. The result showed that the addition of bionanocarbon into the matrix increased the density of both modified fibre composites. The enhancement in density value with the loading of bionanocarbon was probably due to the filler’s nanosize in the low density of the matrix [52]. The carbon nanostructure probably filled up possible hollow or void space in the composites, resulting in a compacted product. It means that the composite mass increased with the bionanocarbon loading while still occupying the same volume, which results in a higher density [53].

**Figure 8:**
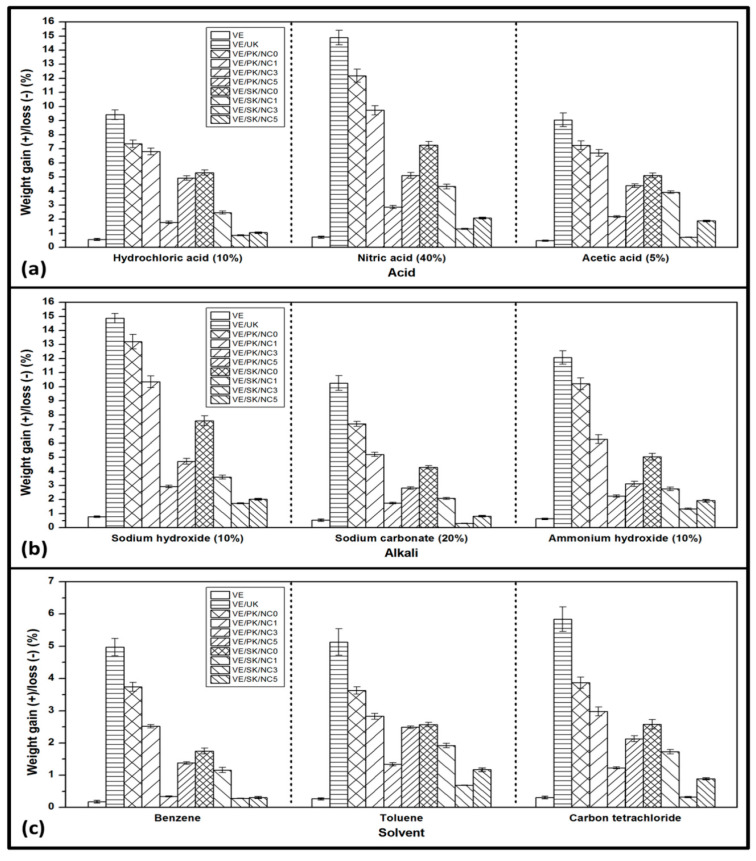
Chemical resistance properties of nanocomposites (**a**) acids resistance, (**b**) alkalis résistance, and (**c**) solvents resistance.

Compatibility of the composites to chemical exposure can be identified through a chemical resistance analysis. The investigation of the composites was conducted to evaluate their performance whether they can properly be applied to make them resistant to chemicals. Figure 8 shows the weight gain/loss of the chemical resistance test for the obtained composites. The figure shows that the obtained composites did not lose weight; hence, erosion does not seem to occur [54]. It was observed in comparative terms between the neat composite and reinforced bionanocomposites at different bionanocarbon loading. Succinylated fibre composites provided better chemical resistance properties compared to propionylated fibre composites. With the incorporation of bionanocarbon, a considerable decrease in acids was achieved optimally at 3% bionanocarbon loading. A similar tendency was observed for the effect of alkalies and solvents resistance on bionanocomposites. This indicated that a firmly embedded formation impacted nanofiller-matrix interaction that enhanced the surface properties of the nanocomposites in terms of chemical exposure. In addition, the hydrophilicity of Kenaf fibre was responsible for the increase in composites’ weight gain. The addition of bionanocarbon probably reduced the hydrophilicity of the composite structure.

##### Mechanical Properties

Mechanical properties of the obtained composites, i.e., tensile strength, tensile modulus, elongation at break, tensile toughness, flexural strength, flexural modulus, flexural toughness, and impact strength, are presented in Table 1. It was aimed to identify the mechanical performance of the neat vinyl ester composite, unmodified Kenaf fibre-based composite, propionylated Kenaf fibre-based composite, and succinylated Kenaf fibre-based composite. The value of tensile strength and tensile modulus increased with the addition of bionanocarbon in the fibre-matrix interface in the modified Kenaf fibre-based composites compared to composites without bionanocarbon. The filler loading up to 3% considerably resulted in the decrease in void content in the fibre-matrix interaction that raises the compatibility of the composite structure. The results showed higher tensile properties of the composites containing succinylated fibre compared to propionylated fibre derived composites. This was probably attributed to greater interfacial fibre-matrix adhesion properties as succinic anhydride modification of fibres allows better mechanical interlocking due to higher dipolar interactions between anhydride and cellulosic hydroxyl groups (–OH) on the natural fibre [55]. Superior interfacial bonding promotes high resistance to fibre pullout without or less rupturing, resulting in enhancement of bionanocomposites’ tensile properties [56]. Improved compatibility was obtained at 3% bionanocarbon loading for the propionylated, and succinylated Kenaf fibre reinforced composites with equivalent tensile strength and tensile modulus of 75.28 ± 1.28 and 92.47 ± 1.19 MPa, and 2.63 ± 0.08 and 3.16 ± 0.08 GPa, respectively. However, weak dispersion and poor interfacial interaction corresponded to decreased tensile strength in the bionanocomposites, as indicated by the composites having beyond 3% bionanocarbon loading.

Similarly, results of tensile toughness, flexural toughness, flexural strength, impact strength, and flexural modulus were increased with the incorporation of bionanocarbon as identified in succinylated Kenaf fibre reinforced composites had better compatibility compared to control and propionylated Kenaf fibre-based composites. In contrast, the decrease in percent elongation at break was observed for composites containing the nanofiller. The succinic anhydride-based composites with 3% bionanocarbon loading showed the lowest percent elongation at break, indicating that an enhancement of compatibility of the fibre-matrix structure was obtained as the value of elongation at break decreased. The mechanical properties obtained in this study were relatively greater than those reported in the recent literature [57], which used bionanofiller in Kenaf fibre/thermoset composites. To recapitulate, a significant enhancement of the mechanical properties of modified fibre composites as bionanocarbon increased was probably due to hydrogen bonding within the composite structure, which results in a cohesive chain. This could be attributed to the surface functionality of the hydroxyl and carboxyl groups in the nanofiller-fibre-matrix interaction.

##### Morphological Analysis

**Figure 9:**
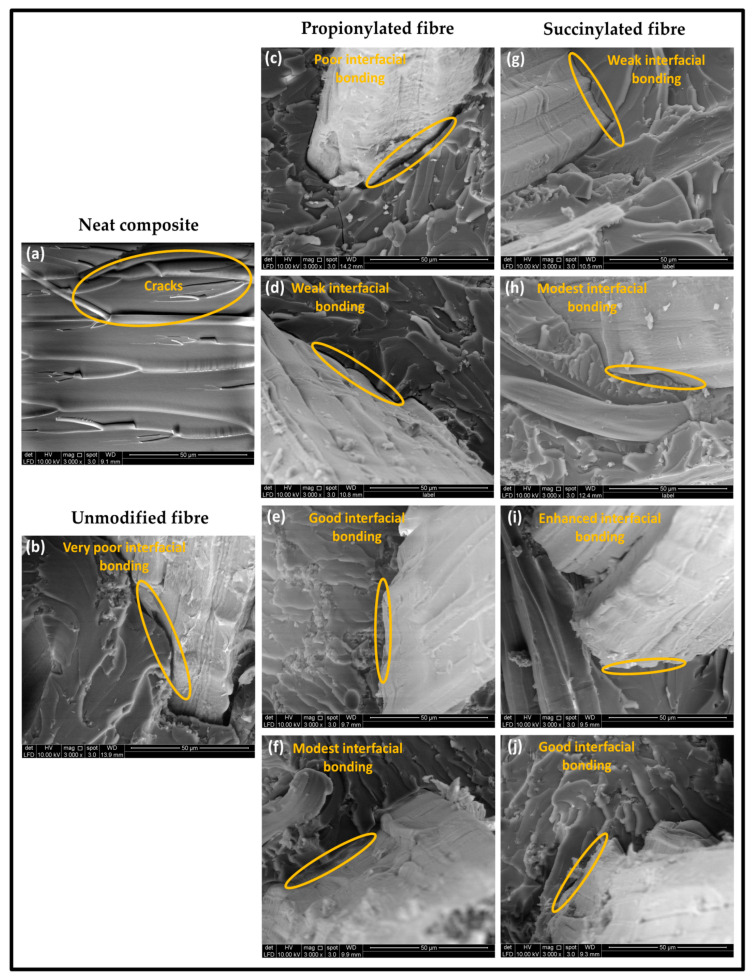
FESEM micrographs of tensile fracture sample of (**a**) VE, (**b**) VE/UK, (**c**) VE/PK/NC0, (**d**) VE/PK/NC1, (**e**) VE/PK/NC3, (**f**), VE/PK/NC5, (**g**) VE/SK/NC0, (**h**)VE/SK/NC1, (**i**) VE/SK/NC3, and (**j**) VE/SK/NC5.

Figure 9 shows the morphological properties of the obtained composites after the tensile test. The neat vinyl ester displayed the brittle features as cracks appeared on the surface (Figure 9a). The unmodified Kenaf fibre composite displayed a very weak interfacial fibre-matrix bonding due to the crack and gap across the boundaries, indicating apparent fracture within the interface. This showed that unmodified Kenaf fibre and the matrix have poor interfacial bonding and weak fibre-matrix adhesion (Figure 9b). As shown in Figure 9c, the propionylated fibre in the composite without bionanocarbon provided a poor interfacial bonding. As bionanocarbon was introduced, compatibility was enhanced due to the well-embedded fibres filling the nanofiller in the available voids within the Kenaf fibre-vinyl ester interface. An improvement in the interfacial fibre-matrix interaction was consequently achieved, as indicated in Figure 9e,f. However, succinic anhydride modification provided a better enhancement of interfacial fibre-matrix bonding than propionylated fibre composite, as shown in Figure 9g. A significant enhancement of compatibility was observed with the introduction of bionanocarbon in the composites (Figure 9h–j). This was probably due to its highly homogenous nanofiller, uniform dispersibility as superior reinforcing filler, resulting in a cohesive chain in the interaction of fibre-matrix [21]. In summary, succinylated fibre composite with 3% bionanocarbon presented an enhanced interfacial adhesion with other constituents in the matrix according to the SEM micrograph of the tensile fracture.

##### Thermal Properties

**Figure 10:**
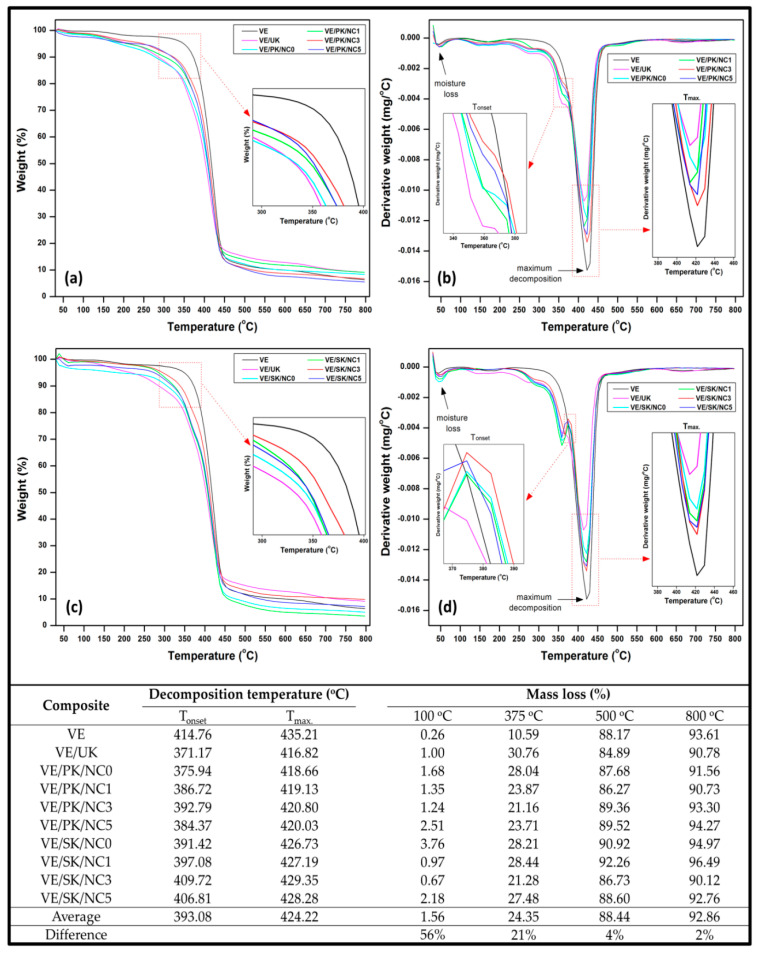
Thermal properties of (**a**) TGA profile of propionylated fibre nanocomposite, (**b**) DTG profile of propionylated fibre nanocomposite, (**c**) TGA profile of succinylated fibre nanocomposite, (**d**) DTG profile of succinylated fibre nanocomposite, and decomposition temperature and mass loss data of the nanocomposite.

The thermal properties of the obtained composites were investigated from the temperature profile. Figure 10 exhibits the composites’ thermogravimetric analysis (TGA) and derivative thermogravimetric (DTG) analysis indicated by the weight loss and derivative weight loss, respectively. The TG curve showed that the evaporation of adsorbed moisture from 40 °C to 100 °C was an indication of initial weight loss for all specimens. The second stage shown in the TG feature was also the maximum degradation stage of all composite samples that occurred from 350 °C to 450 °C corresponded to the thermal degradation of Kenaf fibre, and vinyl ester bionanocarbon can withstand at the high temperatures [58]. It was observed that the weight loss decreased with the increase of bionanocarbon loading in the propionylated Kenaf fibre bionanocomposites (Figure 10a). As shown in Figure 10b, composite containing 3% bionanocarbon loading provided higher decomposition temperature compared to those with reinforcing materials. However, succinylated Kenaf fibre composites showed greater thermal resistance than the composites containing Kenaf fibre with propionic modification (Figure 10c). The weight loss decreased as the bionanocarbon increased, although it was observed lower than the propionylated composites. A higher degradation temperature was found at succinylated composite with 3% nanofiller loading (Figure 10d). The obtained result showed that succinylated-based composites consisted of a great embedded structure incorporating bionanocarbon that could form a great bonding between Kenaf fibre and vinyl ester. Finally, bionanocarbon’s dehydroxylation was observed at a temperature from 500 °C to 800 °C. The introduction of bionanocarbon into the composites enhanced thermal stability, as identified from the plot. Its great distribution in the Fibre-matrix structure corresponded to reducing the composites’ weight loss regardless of its percentage loading.

A recapitulation of numerical data derived from the thermogravimetric analysis is rendered in Figure 10. The initial temperature of degradation (T_on_) and the maximum temperature of degradation (T_max_) was shifted to a higher temperature as the nanofiller loading increased in the composites up to 3% of both Kenaf fibres and decreased beyond the optimum nanofiller loading. The addition of 3% bionanocarbon in propionylated and succinylated composites shifted the T_on_ from 375.94 °C to 392.79 °C and 391.42 °C to 409.72 °C, respectively. Similarly, the T_max_ of propionylated and succinylated composites shifted from 418.66 °C to 420.80 °C and from 426.73 °C to 429.35 °C, respectively. Composites containing succinylated fibre provided greater thermal stability since the T_on_ and T_max_ were higher than those having propionylated fibre. Such enhancement could be attributed to the stronger intermolecular interactions promoted by incorporating the bionanocarbon that could enhance the compatibility of the composite network by occupying the empty voids in the composite interface [59,60]. The mass loss at 375 °C indicated that they decreased as the nanofiller loading increased. The composite samples having 3% bionanocarbon were comparatively lower than composites with other percentage fillers loading; however, the neat vinyl ester had the lowest. In conclusion, the succinylated bionanocomposite exhibited optimum thermal stability with 3% bionanocarbon with the lowest percent mass loss at the extreme temperatures of 500 °C to 800 °C.

## 4. Conclusions

Kenaf fibre was successfully modified using propionic and succinic anhydrides. The anhydrides modification provided hydrophobicity and removed the impurities of Kenaf fibre, leading to greater interfacial fibre-matrix interaction. The modified fibres significantly enhanced the functional properties of bionanocomposites compared to unmodified fibre and vinyl ester itself. However, succinylated fibre-based bionanocomposites were comparatively superior to propionylated fibre in enhancing the physical, chemical resistance, mechanical, and thermal characteristics. Bionanocarbon presented better compatibility in fibre-matrix structure with the optimum loading at 3%. The incorporation across the fibre-matrix interface filled the available voids that could enhance interfacial interaction in the bionanocomposites due to its negative surface charge and well-dispersed. In this study, the highest tensile, flexural, and impact strengths of nanobiocomposite were 92.47 ± 1.19 MPa, 108.34 ± 1.40 MPa, and 8.94 ± 0.12 kJ m^−2^, respectively.

Meanwhile, the initial and maximum degradation temperatures for the optimised nanobiocomposie were 409.72 °C and 429.35 °C, respectively. Succinylated Kenaf fibre incorporated with OPS-based bionanocarbon shows great prospect as reinforcing materials in vinyl ester matrix to form bionanocomposites with better functional properties. Hence, the produced bionanocomposite has high potential in industrial-scale applications, including furniture, packaging, storage, and automobile interior.

## Figures and Tables

**Table 1:** Mechanical properties of unmodified, propionylated, and succinylated Kenaf fibre reinforced bionanocomposite.

Composites	Tensile Strength (MPa)	Tensile Modulus (GPa)	Elongation at Break (%)	Tensile Toughness (MPa)	Flexural Strength (MPa)	Flexural Modulus (GPa)	Flexural Toughness (GPa)	Impact Strength (kJ·m^−2^)
VE	31.64 ± 0.82	0.74 ± 0.03	8.82 ± 0.41	42.15 ± 0.36	36.04 ± 1.27	2.63 ± 0.14	42.56 ± 1.70	2.15 ± 0.08
VE/UK	42.79 ± 1.40	1.27 ± 0.06	7.83 ± 0.25	72.21 ± 1.53	51.84 ± 1.26	3.62 ± 0.11	53.09 ± 0.85	3.61 ± 0.18
VE/PK/NC0	54.28 ± 1.27	2.31 ± 0.11	6.80 ± 0.19	82.29 ± 1.06	75.83 ± 1.14	6.13 ± 0.05	64.20 ± 1.39	5.02 ± 0.17
VE/PK/NC1	63.87 ± 1.34	2.44 ± 0.09	5.48 ± 0.16	87.04 ± 0.98	81.95 ± 1.62	7.18 ± 0.11	72.36 ± 0.89	5.37 ± 0.15
VE/PK/NC3	75.28 ± 1.28	2.63 ± 0.08	5.10 ± 0.19	92.49 ± 1.63	94.73 ± 1.32	8.27 ± 0.26	82.75 ± 0.81	6.59 ± 0.14
VE/PK/NC5	71.38 ± 1.43	2.52 ± 0.05	5.27 ± 0.12	90.38 ± 0.94	89.70 ± 1.26	7.93 ± 0.18	78.61 ± 1.77	6.12 ± 0.18
VE/SK/NC0	65.91 ± 1.16	2.57 ± 0.19	5.38 ± 0.11	88.72 ± 0.85	82.35 ± 1.60	7.42 ± 0.16	74.29 ± 1.38	5.51 ± 0.16
VE/SK/NC1	81.72 ± 1.26	2.81 ± 0.09	5.06 ± 0.17	95.81 ± 1.24	96.36 ± 1.62	8.39 ± 0.06	84.03 ± 0.75	6.90 ± 0.12
VE/SK/NC3	92.47 ± 1.19	3.16 ± 0.08	4.82 ± 0.12	106.71 ± 1.79	108.34 ± 1.40	9.56 ± 0.08	93.49 ± 0.97	8.94 ± 0.12
VE/SK/NC5	87.40 ± 1.82	2.93 ± 0.05	4.95 ± 0.16	102.48 ± 0.76	104.59 ± 1.07	9.14 ± 0.11	88.29 ± 1.37	8.35 ± 0.18

## Data Availability

Not applicable.
